# TNF-Α May Mediate Inflammasome Activation in the Absence of Bacterial Infection in More than One Way

**DOI:** 10.1371/journal.pone.0071477

**Published:** 2013-08-07

**Authors:** Susana Álvarez, Ma Ángeles Muñoz-Fernández

**Affiliations:** 1 Laboratorio InmunoBiología Molecular, Hospital General Universitario Gregorio Marañón, Madrid, Spain; 2 Instituto de Investigación Sanitaria Gregorio Marañón, Madrid, Spain; 3 Networking Research Center on Bioengineering, Biomaterials and Nanomedicine, Madrid, Spain; Ecole Polytechnique Federale de Lausanne, Switzerland

## Abstract

Members of the mammalian nucleotide binding domain, leucine-rich repeat (LRR)-containing receptor family of proteins are key modulators of innate immunity regulating inflammation. To date, microbial pathogen-associated molecules and toxins have been identified as key triggers of activation of inflammasomes. However, recently, environmental, and neurodegenerative stimuli have been identified that lead to IL-1β release by means of inflammasomes. IL-1β plays a crucial role during brain inflammation, and caspase-1 appears to be a key modulator of IL-1β bioactivity and the consequent transcriptional regulation of gene expression within the brain during inflammation. We show here that exposure of a human neuroblastoma cell line (SK-N-MC cells) to TNF-α promotes ROS-mediated caspase-1 activation and IL-1β secretion. The involvement of NF-κB in the regulation of IL-1β synthesis is investigated through specific inhibition of this transcription factor. The effect of TNF-α was abolished in the presence of ROS inhibitors as NAC, or DPI. Remarkably, SK-N-MC cells do not respond to ATP stimulation in spite of P2X_7_R expression. These results provide a mechanism by which danger signals and particulate matter mediate inflammation via the inflammasome in the absence of microbial infection.

## Introduction

Inflammasomes are multiprotein complexes responsible for the activation of caspase-1, and caspase-5 proteases required for processing and activation of proinflammatory cytokines IL-1β and IL-18 [1], [2]. To date, four bonafide inflammasomes named by the PRR that regulates their activity have been identified: the NALP1, NALP3, NLRC4 and AIM2 inflammasomes. With the exception of AIM2, the other inflammasomes contain a PRR that belongs to the Nod-like receptor (NLR) family [3].

The NLR proteins are commonly organized into three domains, a C-terminal leucine-rich repeat (LRR) domain, an intermediate nucleotide binding and oligomerization domain (NOD, also called NACHT domain) and a N-terminal pyrin (PYD), caspase activation and recruitment domain (CARD) or a baculovirus inhibitor of apoptosis repeat domain (BIR). The LRR domains of these proteins are hypothesized to interact with putative ligands and play a role in auto-regulation of these proteins. The NACHT domain can bind to ribonucleotides, which regulates the self-oligomerization and inflammasome assembly [4]. The N-terminal domains, which mediate protein-protein interactions with downstream signaling intermediates, are also used to subcategorize the NLR proteins. A group of 14 NLRP proteins in humans carry a PYD domain. NOD1 (NLRC1), NOD2 (NLRC2) and NLRC4 (also called IPAF) instead express an N-terminal CARD domain while NAIP5 has a BIR domain at the N-terminus [5].

It has been previously described that caspase-1 is activated after a 2-step process, in which muramyl dipeptide binding to NALP1 first induces a conformational change of the protein, which is secondarily followed by ATP binding to mediate NALP1 oligomerization [6]. Upon oligomerization of NALP1, caspase-1 monomers associate with NALP1 oligomers, which results in protease activation, presumably via an induced dimerization mechanism [7].

NALP1 is highly expressed in the brain, whereas no signal is found for NALP3 [8]. Bearing in mind the NALP1 expression in macrophages, it was expected that microglial cells would be the brain cells expressing NALP1. Moreover, microglia cells have been reported to secrete IL-1β, at least in culture [9]. Unexpectedly, however, neurons, in particular pyramidal ones, and oligodendrocytes stained positive for NALP1, whereas microglia were negative [8].

Caspase-1 processes a number of cellular substrates, which is a prerequisite for the induction of an inflammatory response. Most notably, caspase-1 converts pro-IL-1β, and pro-IL-18 into their mature biologically active cytokine forms [10], [11]. Moreover, caspase-1 is required for the release of a number of pro-inflammatory molecules, which are not necessarily caspase-1 substrates, including IL-1α [12]. In addition to its pro-inflammatory effects, excessive activation of caspase-1 leads to a form of cell death called pyroptosis, with characteristics of both apoptosis and necrosis [13]. Thus, pharmacological strategies for regulating caspase-1 represent attractive approaches for disease intervention.

IL-1 is an important initiator of the immune response, playing a key role in the onset and development of a complex hormonal and cellular inflammatory cascade. Elevated IL-1β has been detected in the brain parenchyma within the early hours after brain injury in both humans and rodents [14]. Nonetheless, IL-1 has been documented to play a role in neuronal degeneration. In astrocytes, IL-1 induces IL-6 production, stimulates iNOS activity [15], and induces the production of macrophage colony stimulating factor (MCSF). In addition, IL-1 enhances neuronal acetylcholinesterase activity, microglial activation and additional IL-1 production, astrocyte activation, and expression of the beta-subunit of S100 protein (S100*β*) by astrocytes, thereby establishing a self-propagating cycle [16].

The molecular mechanisms of how inflammasomes can recognize such a diverse array of activators and the role of transcriptionally active signaling receptors for their activation are controversial and mechanistically poorly understood.

It has been further suggested that reactive oxygen species (ROS) may be involved in this process. Depletion of the p22phox subunit of the ROS-generating NADPH complex in the human monocytic cell line THP-1 results in reduced IL-1β processing in response to asbestos, but not MSU crystals [17]. Moreover, high extracellular potassium opens pannexin channels leading to caspase-1 activation in primary neurons and astrocytes [18]. The effect of K^+^ on pannexin 1 channels is independent of membrane potential, suggesting that stimulation of inflammasome signaling is mediated by an allosteric effect. The activation of the inflammasome by K^+^ is inhibited by the pannexin 1 channel blocker probenecid, supporting a role of pannexin 1 in inflammasome activation [18]. On the other hand, ATP activates the P2X_7_R cation channel, which induces potassium efflux and causes the recruitment of the pannexin-1 channel that amplifies this response [19].

It is becoming increasingly evident that neuroinflammation plays a crucial role in the development and progression of many neurodegenerative diseases. Moreover, activated microglia secrete a variety of inflammatory mediators including cytokines as tumor necrosis factor-α (TNF-α) that promote the inflammatory state. Therefore, the investigation of the regulatory or modulatory mechanisms for brain maturation and release of IL-1β may provide therapeutic clues for neuroinflammatory/neurodegenerative diseases.

Here we demonstrate that the proinflammatory cytokine TNF-α triggers the generation of short-lived ROS in SK-N-MC cells, and treatment with various ROS scavengers’ blocks inflammasome activation, and by the way IL-1β production. Moreover, interestingly, TNF-α could generate both signals necessary for IL-1β production that is priming via NF-κB and maturation via caspase-1.

Therefore, a deeper understanding of inflammasome-mediated innate immune responses is warranted toward the development of therapeutic strategies for infectious disease.

## Methods

### Cell Culture and Treatments

The SK-N-MC (from ATCC HTB10), cell line was routinely grown in RPMI 1640 (Biochrom KG Seromed, Berlin, Germany) containing 10% heat-inactivated fetal calf serum, 1% penicillin/streptomycin, and 2 mM L-glutamine (ICN Pharmaceuticals) at 37°C and 5% CO_2_. HEK-Blue™ IL-1β cells (InvivoGen, San Diego, USA) were cultured in Dulbecco’s modified Eagle’s medium (Gibco, Rockville, MD, USA) supplemented with 30 µg/ml blasticidin and 100 µg/ml Zeocin.

RhTNF-α was from Promega (Promega Corporation, WI, USA). LPS, and actinomycin D were purchased from Sigma (St. Louis, MO, USA). N-Ac-Tyr-Val-Ala-Asp-CMK (Ac-YVAD-CMK) (selective caspase-1/ICE inhibitor), and BAY 11-7082 were from Cayman Chemical, Ann Arbor, MI, USA). ATP, and Muramyl dipeptide (MDP) were from InvivoGen (San Diego, CA, USA). Reagents were dissolved following manufacturer’s instructions, and final drug dilutions were prepared fresh in complete tissue culture medium and warmed to 37°C before use.

### Western Blot Analyses

Cells were exposed to different stimuli, washed with phosphatebuffered saline (PBS) and lysed with buffer lysis. Protein contents were measured using bicinchoninic acid method (BCA protein assay) according to the manufacturer’s instructions (Pierce, Rockford, IL, USA). Samples were separated into a 10–15% SDS polyacrylamide gel and blotted onto a polyvinylidene fluoride membrane (Millipore, Bedford, MA, USA) by semidry transference blotting. Membranes were blocked overnight at 4°C using Rotiblock (Roth, Karlsruhe, Germany) before incubation with the primary antibody. Rabbit polyclonal antibodies to P2X_7_ (H-265) (Santa Cruz Biotechnology, Inc. CA, USA), cleaved IL-1β (Cell Signaling Technology, Inc, Beverly, MA, USA), and mouse monoclonal antibodies to caspase-1, and NALP1 (Santa Cruz Biotechnology, Inc. CA, USA), were used as primary antibodies as appropriate. Membranes were washed and incubated with horseradish peroxidase-conjugated secondary antibody (Amersham, GE Healthcare, UK; 1∶10000) for 1 h at room temperature. Proteins were detected using the Immun-Star Western C Kit (Bio-Rad Laboratories, Hercules, CA, USA). In all cases, equal amounts of total protein were analyzed across groups. α-tubulin (Sigma, St. Louis, MO) was used as internal control to validate the amount of protein loaded onto the gels. For quantitation, the pixel intensity for each band was determined using the Image/J program, and normalized to the amount of α -tubulin.

### Caspase-1 Assay

Caspase-1 activity was measured using the Caspase-1 Fluorometric Assay Kit (Abcam, CB4 0FL, UK) according to the manufacturer’s protocol. The assay is based on detection of cleavage of substrate YVAD-AFC (AFC: 7-amino-4-trifluoromethyl coumarin). YVAD-AFC emits blue light (400 nm); upon cleavage of the substrate by caspase-1 free AFC emits a yellow-green fluorescence (505 nm), which can be quantified using a fluorometer or a fluorescence microtiter plate reader. Comparison of the fluorescence of AFC from a treated sample with an untreated control allows determination of the fold increase in caspase-1 activity. Data are expressed as fold-increase over unstimulated cells and are the mean and SEM of at least three separate experiments.

### Quantification of IL-1β

SK-N-MC cells were either left untreated or treated for 24 h with different stimuli. Next, levels of IL1-β in cell-free supernatants were determined through the use of HEK-Blue™ IL1-β cells according to the manufacturer’s protocol (InvivoGen, San Diego, CA). 20 µl of each sample was added to a flat bottom 96-well plate containing 50,000 HEK-Blue™ IL1-β cells per well and incubated at 37°C for 24 h. Forty µl of induced HEK-Blue™ IL-1β supernatant were then collected and incubated with 160 µl of QUANTI-Blue (InvivoGen, San Diego, CA, USA) per well for 1 h at 37°C. Quantification of the activation of the IL1-β pathway is made through the inclusion of a reporter gene expressing a secreted form of embryonic alkaline phosphatase under the control of an NF-κB-inducible promoter. Secreted embryonic alkaline phosphatase levels were determined using a spectrophotometer at 650 nm and compared with a standard curve made by serial dilutions of the IL-1β protein.

### Detection of Intracellular H_2_O_2_ and Assessment of Mitochondrial O_2_–

The H_2_O_2_–sensitive fluorescence probe carboxy-2′,7′-dichlorodihydrofluorescein diacetate (DCFH-DA) (Sigma, St Louis, MO, USA) was used to assess the generation of intracellular H_2_O_2_. To detect mitochondrial O_2_–, MitoSOX Red (Invitrogen Corporation, Frederick, MD, USA) a mitochondrion-specific hydroethidine-derivative fluorescent dye, staining was carried out according to the manufacturer’s instructions. In order to understand the role of ROS formation in the assembly of inflammasome, ROS inhibitor, diphenyleneiodonium (DPI), or apocynin (Sigma, St. Louis, MO, USA) were used. Briefly, SK-N-MC cells were incubated for 4 h with 10 µM DPI, and 5 µM apocynin or 25 µM N-acetylcysteine (NAC) followed by stimulation with TNF-α for 24 h. For the estimation of intracellular ROS, the supernatant was replaced with DCFH-DA (10 µM) and incubated for 1 h at 37°C. The wells were then washed with freshly prepared Hank’s balanced salt solution and fluorescence intensity measured using a fluorescence microplate reader. For measuring mitochondrial ROS, cells were incubated with MitoSOX 5 µM for 10 min at 37°C. After washing, mitochondrial ROS levels were measured using CellQuest Pro software on FACScan flow cytometer (Becton Dickson, San Jose, CA, USA) (excitation, 488 nm; emission, 530 nm), or by a fluorescence microscope (LEICA AOBS-TCS-SP2 system). The data are presented as fold change in the mean intensity of MitoSOX fluorescence compared with the respective controls.

### Confocal Analysis

Cells were fixed in PBS (pH 7.4) containing 3.7% para-formaldehyde and 0.025% glutaraldehyde for 10 min. Fixed cells were permeabilized in PBS containing 0.1% Saponine for 10 min. After two washes in PBS, cells were incubated with 1% bovine serum albumin in PBS (pH 7.4) for 20–30 min. Immunofluorescence staining was performed with polyclonal anti-IL-1β followed by secondary antibody conjugated with Texas Red. The polyclonal IL1-β antibody used in fluorescence microscopy recognizes both pro-IL-1β and mature IL1-β (Santa Cruz Biotechnology, Inc. CA, USA). DAPI (1 µg/ml; Alexis Biochemicals) was applied to label nuclei. Confocal laser scanning microscopy was performed with a LEICA AOBS-TCS-SP2 system. Separate images were taken in the corresponding channels, and merge images were composed. Image acquisition and data processing for all the samples were performed under the same conditions.

### Intracellular Ca^2+^ Concentration Assay

Intracellular Ca^2+^, [Ca^2+^]i, concentrations were measured using the compound Fluo-4AM (Invitrogen Corporation (Frederick, MD, USA) according to manufacturer’s instructions. After a washing step in PBS-Ca^2+^ the cells were resuspended in the appropriate buffer in accordance to the different conditions and kept at 37°C until analysis. For condition (a) a PBS+Ca^2+^ buffer was used containing 1 mM sodium pyruvate and 25 mM HEPES. This condition allows changes of [Ca^2+^ ]i originating from the release of Ca^2+^ from the intracellular stores and from the entry of Ca^2+^ extracellular into the cytosol and thus corresponds to the in vivo condition. For condition (b) Ca^2+^ entry is prevented by the use of a PBS-Ca^2+^ buffer containing 1 mM sodium pyruvate, 25 mM HEPES and 5 mM EGTA.

### Flow Cytometry Measurement of [Ca^2+^]_i_ in SK-N-MC Cells and Addition of Stimuli

Samples were analyzed using the FACScan flow cytometer with an argon laser with fixed output wavelength of 488 nm. Following preparation the samples were divided into two equal volumes and the first aliquot was aspirated during 30 s to determine the baseline fluorescence of the Fluo-4-Ca^2+^-complex. Next, a stimulus, TNF-α (20 ng/ml) or A23187 (2 µM), was added to the second aliquot. The aspiration of the baseline sample was paused, samples were switched and the acquisition was resumed with changes in [Ca^2+^]i being recorded over a 300 s time period.

### Statistical Analysis

Statistical analyses were performed with the program GraphPad Prism. The significant value (P value) for the parameters measured in all assays was calculated using the students test.

## Results

Previously, we found that TNF-α induces caspase-3-independent cell death in SK-N-MC cells, as well as caspase-1 and PYCARD gene expression by microarray analysis, and by using the Ingenuity Pathway Analysis (IPA) system [20]. Again, in the present study and to continue this line of investigation we have used the human neuroblastoma SK-N-MC cell line, chosen in part to determine the toxic effect of TNF-α in these cells, since neuronal SK-N-MC cells lack glutamate receptor subtypes (Gelbard HA, New D, Dzenko K, Unpublished data).

### SK-N-MC Cells Express NALP-1 Protein

Kummer at al described that in the brain, neurons, in particular pyramidal ones, and oligodendrocytes stained positive for NALP1, whereas microglia were negative [8]. However, as our cell model is the cell line SK-N-MC we investigated NALP1 expression in these cells. At the protein level, SK-N-MC cells express NALP1 protein and this expression is not altered after treatment with TNF-α (Figure S1).

### TNF-α Induces Caspase-1 and IL-1β in SK-N-MC Cells

Caspase-1 is itself produced as an inactive zymogen that must be cleaved to generate the active p10 and p20 subunits. Since the secretion of IL-1β requires both the transcription of IL-1β and caspase-1 activation, and having established that SK-N-MC cells express NALP1, we next sought to determine whether TNF-α, would stimulate neuronal cultures to generate a caspase-1 activation.

**Figure 1:**
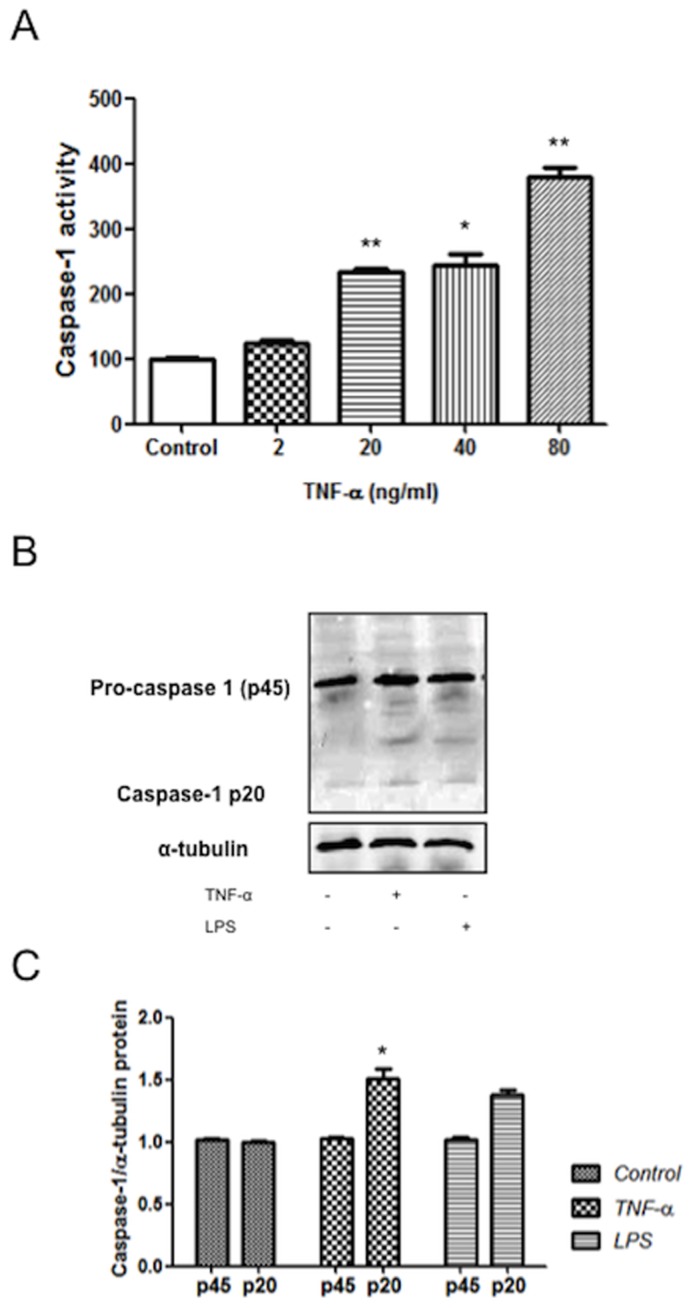
TNF-α induces caspase-1 activation in SK-N-MC cells. A) SK-N-MC cells were stimulated for 24 h with the indicated concentrations of TNF-α. Caspase-1 activity was measured in the cell lysates by a fluorometric assay. B) Caspase-1 processing in SK-N-MC cells stimulated with TNF-α was examined by Western blot. Cells were stimulated for 24 h with TNF-α (20 ng/ml), or LPS (1 µg/ml). C) Graph depicting the results obtained after performing a densitometer analysis of the blots. A, B Immunoblots are representative of three independent experiments. Results are representative of three separate experiments. Quantitative comparisons of the caspase-1 intensities between the control and stimulated cells. Statistical differences in comparison to control values *:p<0.05.; **:p<0.01.

Using caspase-1 activity assay, we observed a significant 2-fold increase in caspase-1 activation with a dose of 20 ng/ml over control cells, and higher with increasing concentrations of TNF-α (Figure 1A). Moreover, to check whether caspase-1 was increased at protein level, we performed Western blot on total cell lysates from TNF-α-stimulated SK-N-MC cells. Stimulated cells increased caspase-1 protein levels as determined by immunoblotting with an Ab that detects endogenous levels of pro-caspase-1 and the caspase-1 p20 subunit (Figures 1B, 1C). An inflammatory signal, LPS, was used as a positive control since it is generally accepted that LPS promotes the synthesis and cytoplasmic accumulation of the inactive precursor (pro-IL-1), and SK-N-MC cells express TLR4 (unpublished results). We confirmed the specificity of the signal by using Ac-YVAD-CMK, a specific caspase-1 inhibitor (data not shown).

**Figure 2:**
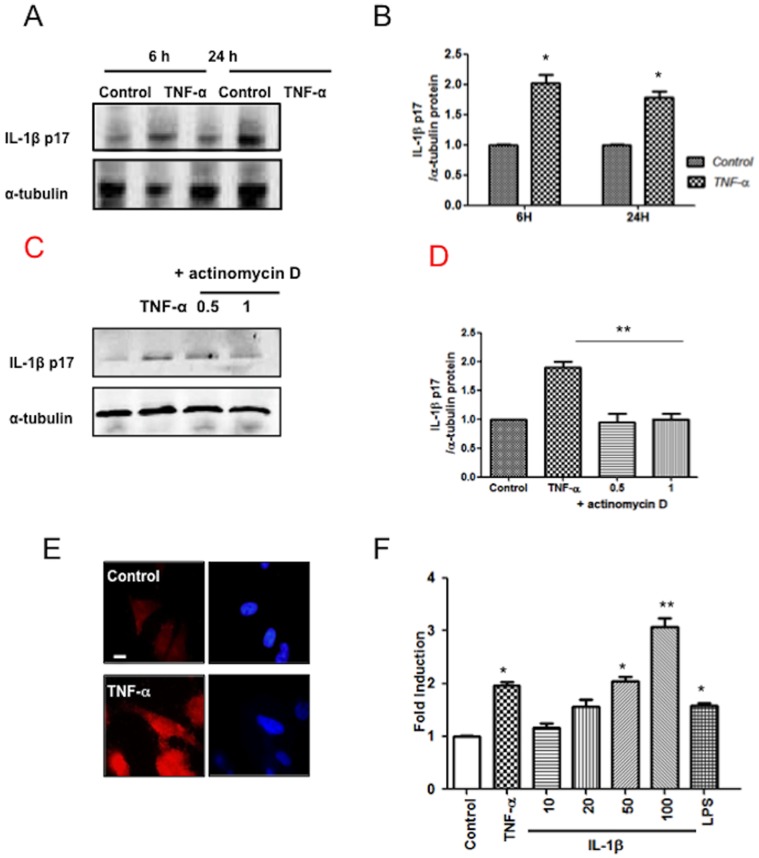
TNF-α activates IL-1β synthesis, and secretion in SK-N-MC cells. A) SK-N-MC cells were stimulated during the indicated time with TNF-α (20 ng/ml). Cell lysates were subjected to SDS-PAGE and immunoblotting with anti-IL-1β (top blot). As loading control, the blots were stripped and incubated with anti-α-tubulin (bottom blot). B) Graph depicting the results obtained after performing a densitometer analysis of the blots. For quantitation, the pixel intensity of each band was normalized to the amount of tubulin to verify uniformity in gel loading. C) IL-1β levels in TNF-α-stimulated cells pretreated or not with actinomycin D at indicated concentrations. D) The graph depicting the results obtained after performing a densitometer analysis of the blots. E) IL-1β intracellular expression by immunofluorescence. Cells were stained with anti-IL-1β monoclonal Ab (red) in control cells (left) and after overnight exposure to 20 ng/ml TNF-α (right), and analyzed by confocal microscopy. Nuclei were visualized by staining with DAPI Bar: 20 µm. F) Stimulation of HEK-Blue™ IL-1β cells by supernatants from SK-N-MC cells stimulated with TNF-α, LPS or directly with recombinant human IL-1β. After 24 h of incubation, SEAP activity was assessed using QUANTI-Blue™ and reading the optical density (O.D.) at 655 nm. The results of at least three independent experiments are shown. The densities obtained from each of the lanes were normalized to the loading control and later to the control sample. Error bars indicate standard error values. Statistical differences in comparison to control values *:p<0.05.; **:p<0.01.

IL-1β is synthesized inside cells as a biologically inactive precursor that is processed by caspase-1 prior to its secretion, and since TNF-α was able to increase caspase-1 activation, we wondered whether also could induce IL-1β synthesis and secretion by SK-N-MC cells. Western blot analysis demonstrated that the p17 subunit, the mature processed form of IL-1β, was induced after TNF-α treatment at both times tested (Figures 2A, 2B).

Furthermore, the secretion of IL-1β after TNF-α treatment was inhibited by Ac-YVAD-CMK, a specific peptide inhibitor of caspase-1, (data not shown).

Treatment of SK-N-MC cells with the transcription inhibitor actinomycin D at concentrations of 0.5, and 1 µM led to reduced IL1-β activation obtained by the stimulation with TNF-α, indicating that protein de novo synthesis was functionally limiting in SK-N-MC cultures (Figures 2C, 2D). We also detected IL-1β protein by confocal analysis, suggesting that the majority of processing of pro- to mature IL-1β in SK-N-MC cells is a cytosolic event (Figure 2E).

Next, we analyzed whether TNF-α could induce cytokine secretion from SK-N-MC cells. To explore whether the increased intracellular pro-IL-1β levels in TNF-α treated cells was caused by reduced IL-1β processing and/or release, we determined the amount of extracellular IL-1β by using the system of HEK-Blue™ IL-1β cells. This system allows detecting bioactive IL-1β by monitoring the activation of the NF-κB and AP-1 pathways. They derive from HEK-Blue™ TNF-α/IL-1β cells in which the TNF-α response has been blocked. Therefore, HEK-Blue™ IL-1β cells respond specifically to IL-1β. SK-N-MC cultures were stimulated with TNF-α at different doses, and supernatants were collected 24 h later and assayed for IL-1β secretion. Higher levels of secreted IL-1β were observed in culture supernatants of treated cells compared with control ones, similar to pattern observed with LPS (Figure 2F). Moreover, we found IL-1β secretion in response to TNF-α directly on HEK-Blue™ IL-1β cells, indicating the specificity of the signal. The release of IL-1β into culture supernatants demonstrates that caspase-1 is activated and functional.

In order to further investigate the mechanisms by which TNF-α may trigger inflammasome activation in SK-N-MC cells, we examined the involvement of some known factors implicated in activation of other inflammasomes as NALP3, such as ROS generation, and reduced cytoplasmic potassium concentration.

### Caspase-1 and IL-1 Activation are Mediated through NOX in SK-N-MC cells after TNF-α Stimulation

**Figure 3:**
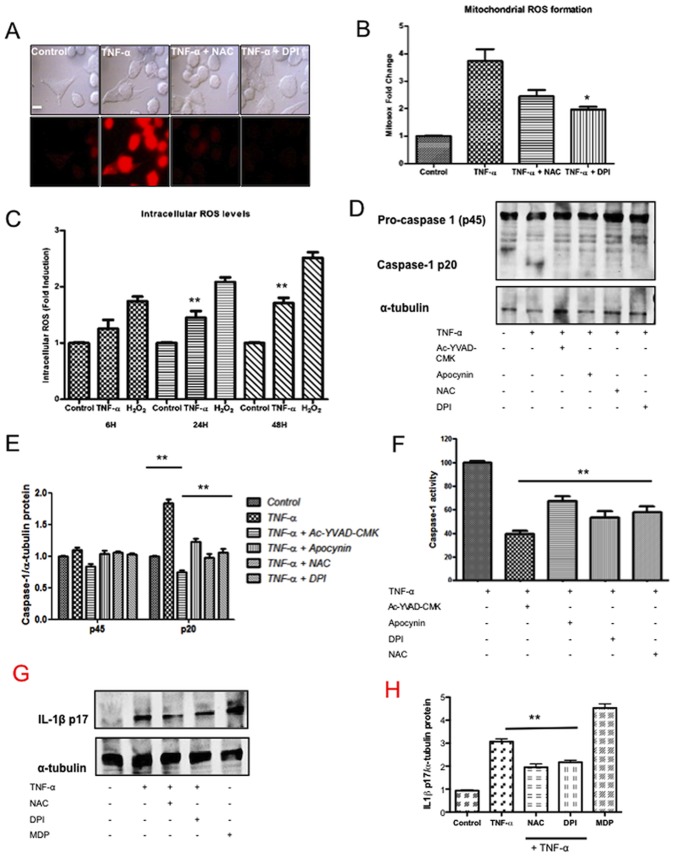
Generation of ROS is critical for caspase-1 activity and subsequent IL-1β. A) SK-N-MC cells were treated with TNF-α for 24 h alone or in combination with DPI or NAC, and mitochondrial ROS formation was measured with MitoSOX by confocal microscopy. Bar: 20 µm. B) Representative histogram of the % MitoSOX positive cells is shown after 24 h of TNF-α stimulation in the presence of 10 µM DPI or 25 µM NAC. Representative of four independent analyses. C) SK-N-MC cells were treated with TNF-α for different times and intracellular ROS levels were measured with the fluorescent probe H_2_DCFDA. H_2_O_2_ was used as positive control of ROS generation. D) TNF-α-stimulated SK-N-MC cells, preincubated for 2 h in the absence or presence of 25 µM NAC, 10 µM DPI or 10 µM apocynin. Gel showing procaspase-1 (p45) and its processed p20 subunit is shown. E) Densitometric analysis of immunoblots from SK-N-MC cultures of caspase-1. F) Caspase-1 activity was measured in the cell lysates of stimulated cell as indicated by a fluorometric assay. Graph represents fold change in caspase-1 activity with respect to untreated cells. G) Immunoblot analysis of the mature (p17) form of IL-1β in extracts of TNF-α-stimulated cells in the presence or absence of of 25 µM NAC, and 10 µM DPI. MDP 1 µg/ml was used as positive control. H) Densitometric analysis of immunoblots of IL-1β. Results represent the means of 2 individual experiments. Error bars indicate standard error values. Statistical differences in comparison to TNF-α condition *:p<0.05.; **:p<0.01.

We observed that following TNF-α stimulation, mitochondrial ROS production increased significantly measured by confocal staining (Figure 3A), as well as by cytometric analysis founding an increase of around 4-fold with respect to the control condition, measured by staining with MitoSOX (a fluorescent indicator of mitochondrial superoxide, O_2_−) (Figure 3B). As expected, upon the addition of the ROS scavenger NAC, and specially the NADPH oxidase inhibitor DPI an important drop in ROS levels by 2-fold was observed with respect to TNF-α alone condition (Figure 3B).

When intracellular ROS levels were measured with DCFH-DA an increase of around 2-fold was found at times of 24 and 48 h (Figure 3C).

Moreover, we determined caspase-1 expression after treatment with TNF-α in combination with several antioxidants, founding a significantly inhibition of levels of caspase-1 protein (Figures 3D, 3E) as well as caspase-1 activity (Figure 3F) after different treatments compared with TNF-α alone. Moreover, IL-1β protein levels were indeed impaired significantly in response to TNF-α when using these inhibitors (Figures 3G, 3H), suggesting that, in our model, ROS production might acts as a stress signal for the inflammasome complex activation. As expected, stimulation with MDP augmented IL-1β levels by neuroblastoma cells.

### K^+^ Efflux and ATP Role in IL-1β Production in SK-N-MC Cells

P2X_7_R may affect neuronal cell death through their ability to regulate the processing and release of IL-1β, a key mediator in neurodegeneration chronic inflammation, and, perhaps, some psychiatric diseases [21]. The best characterized activity of the P2X_7_R is its role in IL-1 release from macrophages and microglia primed with substances such as bacterial endotoxin [22]. ATP is the only known physiological activator of the P2X_7_R [23], [24].

**Figure 4:**
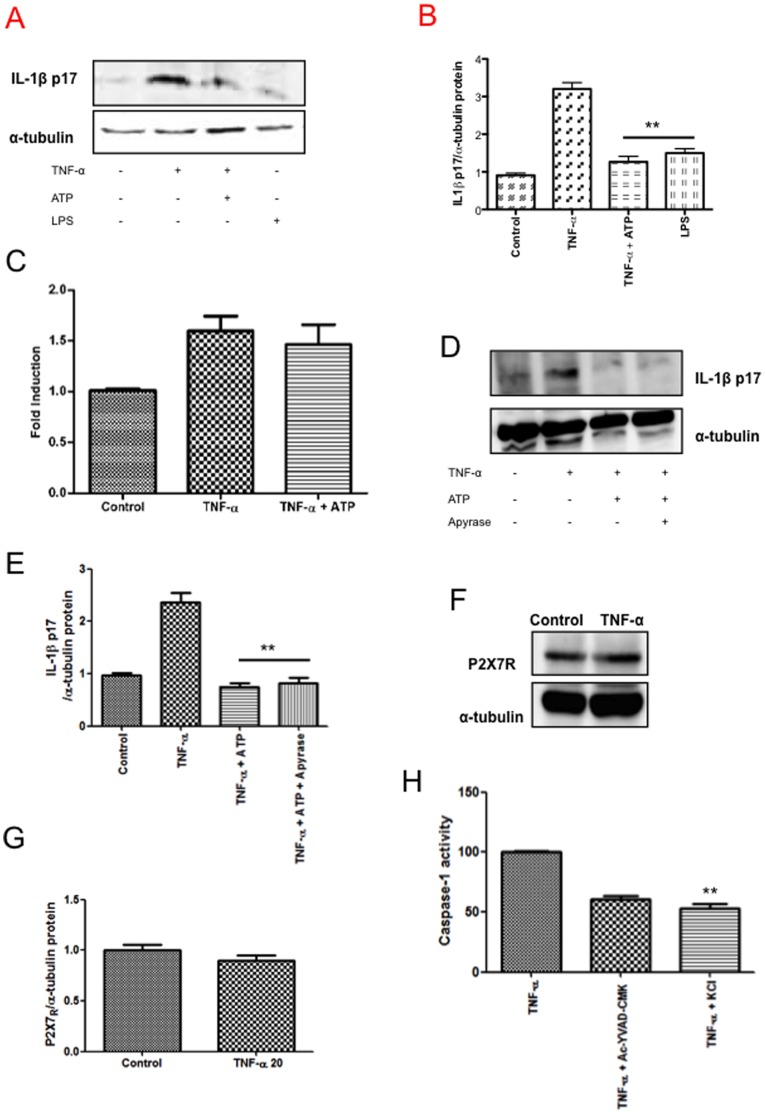
Role of ATP and KCl in TNF-α-induced inflammasome activation. A) SK-N-MC cells were stimulated for 24 h with TNF-α (20 ng/ml), or LPS (1 µg/ml). Where indicated, ATP (5 mM) was added for the last 30 min. Cell lysates were subjected to SDS-PAGE and immunoblotting with anti-IL-1β, or anti-α-tubulin control. B) Graph depicting the results obtained after performing a densitometer analysis of the blots. C) Stimulation of HEK-Blue™ IL-1β cells by supernatants from SK-N-MC cells stimulated with TNF-α. Where indicated, ATP (5 mM) was added for the last 30 min. After 24 h of incubation, SEAP activity of HEK-Blue™ IL-1β was assessed using QUANTI-Blue™. D) IL-1β p17 subunit in stimulated cell in the presence of apyrase (5 units/ml). E) The graph depicting the results obtained after performing a densitometer analysis of the blots. F) Expression levels of the P2X_7_R in SK-N-MC control cells, and after TNF-α treatment (20 ng/ml). G) The graph depicting the results obtained after performing a densitometer analysis of the blots. H) Caspase-1 activity in TNF-α-stimulated in the presence of 20 µM caspase inhibitor Ac-YVAD-CMK or 130 mM extracellular KCl. The results of combining three independent experiments performed in duplicate are shown. Error bars indicate standard error values. Statistical differences in comparison to TNF-α condition **:p<0.01.

To investigate the role of ATP, and P2X_7_R in the secretion of IL-1β induced by TNF-α, SK-N-MC cells were treated with TNF-α alone or in combination with ATP, and protein levels of IL-1β were measured 24 h later. The presence of ATP did not alter levels of IL-1β measured by Western blot (Figures 4A, 4B), and by using the system of HEK-Blue™ IL-1β cells (Figure 4C).

However, it is possible that ATP could be hydrolyzed by alkaline phosphatase, secreted by HEK-Blue™ IL-1β cells and could have competed with QUANTI-Blue for the active site. To resolve this issue we used the ATP degrading enzyme apyrase, founding that levels of IL-1β of treated cells, prior to TNF-α treatment were not significantly different from levels of the cytokine detected in control cells lysates (Figures 4D, 4E). Indeed, although there is a robust expression of P2X_7_R, no variation in protein levels was detected after incubation with TNF-α for 24 h (Figures 4F, 4G).

To test the importance of potassium efflux for the TNF-α-induced inflammasome activation, we treated SK-N-MC neuronal cultures with 130 mM potassium chloride (KCl) for 1 h, and assayed protein lysates for caspase-1 activation. As shown in Figure 4H, we found more than 2-fold reduction in caspase-1 activity with compared with untreated controls.

To definitively discard P2X_7_R and ATP involvement in inflammasome activation by TNF-α, P2X_7_R function was investigated employing a functional calcium flux assay [25], since it has been described that, upon ATP binding, P2X_7_R mediates a slow sustained influx of extracellular calcium that follows an initial fast efflux of intracellular calcium stores mediated by the purinergic receptor P2Y [26]. TNF-α treatment did not lead to a significant up-regulation in Ca^2+^ levels after 300 s after initial cytokine application (Figure S2). Stimulation with ionophore A23187, used as positive control, resulted in a rise in [Ca^2+^]i in SK-N-MC cells in the both conditions tested (with and without EGTA).

This result along with the fact that ATP treatment did not increase IL-1β secretion indicates that inflammasome activation may require potassium efflux as an additional danger signal upon TNF-α stimulation, being independent of the P2X_7_R.

### TNF-α Promotes IL-1β Synthesis through NF-κB Activation

It is generally accepted that secretion of IL-1β occurs in two steps. In the first step, an inflammatory signal, such as LPS, promotes the synthesis and cytoplasmic accumulation of the inactive precursor (pro-IL-1). The second signal triggers caspase-1-mediated processing of pro-IL-1, and secretion of the mature cytokine [27].

**Figure 5:**
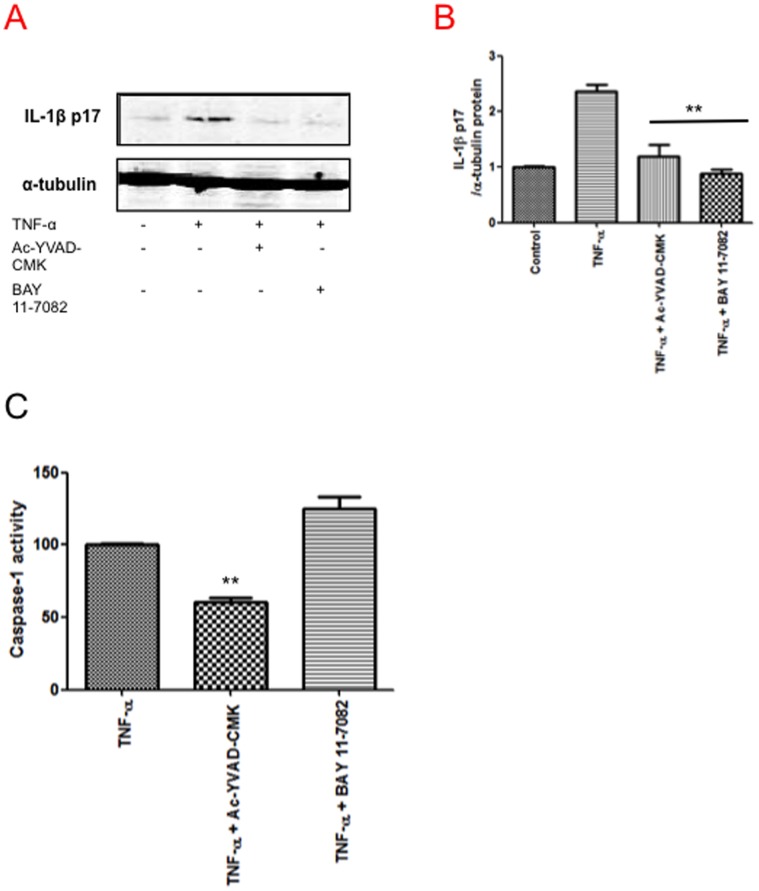
NF-κB involvement in IL-1β activation. A) Immunoblot of IL-1β levels in TNF-α-stimulated cells in the presence of BAY 11-7082 (20 µM). B) The graph depicting the results obtained after performing a densitometer analysis of the blots. C) Caspase-1 activity of stimulated cells preincubated with the NF-κB inhibitor BAY 11-7082. The results of combining two independent experiments performed in triplicate are shown. Error bars indicate standard error values. Statistical difference with respect to TNF-α stimulated condition **:p<0.01.

We confirmed the dependence of TNF-α-induced IL-1β production on NF-κB activation by using BAY 11-7082 (Figure 5A), a drug that inhibits NF-κB activation by targeting the Iκ-B kinase complex [28]. Our results indicate that BAY11-7082 prevents production of pro-IL-1β in a significant manner (Figure 5B).

Interestingly, caspase-1 activation was not altered in the presence of this inhibitor (Figure 5C), indicating that TNF-α might promote inflammasome activation through ROS, as well as proIL-1β synthesis through NF-κB activation.

**Figure 6:**
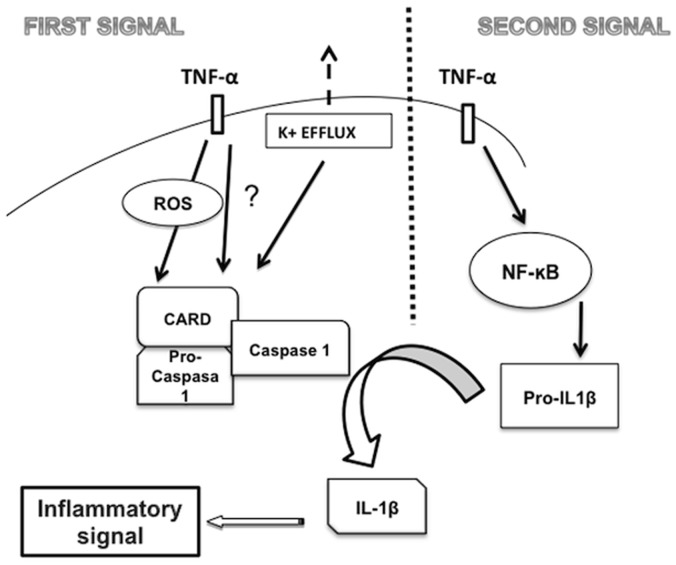
A proposed model outlining how TNF-α induced inflammasome activation in SK-N-MC cells.

The proposed TNF-α-induced inflammasome activation is shown in Figure 6.

## Discussion

The production of IL-1β is subject to complex regulation [29], and consequently, two separate signals are required to yield the active proinflammatory cytokine. First, induction of IL-1β mRNA, for example via stimulation of pattern recognition receptors, is needed for synthesis of proIL-1β protein in cells. A second signal is required for activation of caspase-1, a protease that cleaves proIL-1β into its biologically active secreted form [1]. Caspase-1 activation, in turn, is mediated by cytosolic protein complexes termed inflammasomes, which function in various immune cells [30]. Four distinct inflammasomes have been recognized. These are the NALP1 [2], NALP3 [31], [32], NLRC4 [33], [34], and AIM2 inflammasomes [35], [36], which respond to a variety of different microbial signatures and danger signals [37].

IL-1β is a major inflammatory mediator. Its physiological effects are diverse, including the generation of fever, the recruitment of inflammatory effector cells, the induction of other pro-inflammatory cytokines such as IL-6 and IL-8, and the shaping of T cell responses [38], [39].

Furthermore, IL-1β promotes the secretion of many other cytokines and chemokines and induces the expression of adhesion molecules, endothelin-1, and inducible nitric oxide synthase in endothelial cells [40], [41], [42].

Therefore, a deeper understanding of inflammasome-mediated innate immune responses is warranted toward the development of therapeutic strategies for infectious disease.

NALP1, which is the only NALP protein that has an additional C-terminal CARD domain, was initially identified to form an intracellular multimolecular complex with the adapter protein apoptosis-associated speck like protein (ASC) and the proteins CARDINAL and caspase-5 [2].

Here, we demonstrate that in response to TNF-α there is an activation of caspase-1 *in vitro,* and NALP1 could be the key mediator of this action. The pleiotropic actions of this cytokine are mediated through two distinct cell surface receptors: 55 kDa TNFR1 (p55, or CD120a) and 75 kDa TNFR2 (also called p75, and CD120b). Although it has been described that both TNF-α receptors in the brain are expressed by neurons and glia, receptor distribution varies depending upon activation of either apoptosis or inflammatory regulation, and although the functions of p75 in the brain are still unclear, activation of p55 initiates signals leading to neuronal apoptosis. In this regard, it has been previously determined the presence of p55 TNF-α receptor mRNA by Northern Blot in SK-N-MC cells [43], and flow cytometry studies have demonstrated that p55 TNF receptors were not up-regulated in neuronal SK-N-MC cells after treatment with TNF-α [44].

Several conditions are thought to be required for the activation of the inflammasome, including the interaction of “danger-signaling” molecules with NLRP components, the induction of K^+^ efflux through the P2X_7_R, and the generation of ROS. Redox signaling is also important in the signaling pathways engaged by various inflammatory conditions. ROS production by the PRR, TLR, regulates activation of redox regulated transcription factors (NF-κB and AP-1) and cytokines production [45]. However, the pathway connecting ROS to the inflammasome remains largely unknown.

Our results show that TNF-α significantly increased the intracellular ROS, as assessed by cells stained with DCFH-DA and MitoSOX analysis. Our findings suggest that increased intracellular and mitochondrial ROS generation is a major mechanism by which TNF-α promotes caspase-1 activity in SK-N-MC cells. In addition, because administration of the thiol antioxidant NAC, and DPI agent prevented TNF-α-induced up-regulation of IL-1β levels, it is likely that increased ROS levels have a central role in activation of this cytokine in these cells. ROS may either directly trigger inflammasome assembly or be indirectly sensed through cytoplasmic proteins that modulate inflammasome activity.

Disturbed ionic and neurotransmitter homeostasis contributes to secondary damage induced by traumatic brain injury, spinal cord injury, and stroke [46]. ATP and K+ released from injured cells mediate a variety of toxic metabolic disturbances often leading to cell death. ATP acting on P2X_7_R is a potent stimulus for caspase-1 activation within the NALP3 inflammasome [4]. Apparently the ATP-bound receptor unmasks an ATPase activity of NALP3 with specificity for ATP or dATP. NALP3-catalyzed nucleotide hydrolysis is vital for protein function and is required for NALP3 self-association, interaction with ASC and caspase-1, and IL-1 cytokine release [4]. In contrast, the NALP1 inflammasome exhibits little nucleotide specificity.

Previous studies showed that NALP1 is highly expressed in neurons [47], and in the present study, we report the expression of NALP1 in SK-N-MC cells. In this regard, it is not surprisingly that ATP released after CNS injury activates the NALP1 inflammasome in neurons contributing to increased IL-1β. Indeed, it seems that the addition of extracellular ATP decreased the presence of mature IL-1β. This could be the result of a) more release of mature IL-1β (p17), b) less maturation (i.e. inhibition of inflammasome by eATP), or c) more degradation of mature intracellular IL-1β (p17). There are necessary future studies to determine exactly the routes involved in this effect.

According with this, our data suggest that TNF-α-dependent IL-1β release does not involve activation of this purinergic receptor, since it is not affected by ATP-hydrolyzing enzymes such apyrase. However, we cannot rule out the possibility that inflammasomes present in other CNS cell types are activated by ATP, thus contributing to increased IL-1β production following injury. Although proinflammatory cytokines and bacterial products up-regulate P2X_7_R expression and increase its sensitivity to extracellular ATP [48], [49], we did not find variations in P2X_7_R expression levels in stimulated over control cells.

Even the role of Ca^2+^ on IL-1β secretion is controversial, since it has been described that an increase in [Ca^2+^]i induces IL-1β secretion [50], [51], [52], Walev *et al.* suggest that Ca^2+^ influx inhibits both processing and release [53]. Thus, when we wanted to check whether variations in Ca^2+^ levels would influence in inflammasome activation, founding that in TNF-α-stimulated cells there was no changes in intracellular calcium concentrations.

It is interesting to note that due to the model used in this study, as neuroblastoma cells, in the complex interplay between malignant cells and their microenvironment, caspase-1 activation complexes have contrasting roles. Inflammasomes may operate at the cell-autonomous level to eliminate malignant precursors through programmed cell death or, conversely, may stimulate the production of trophic factors for cancer cells and their stroma. In inflammatory cells, caspase-1 activation can fuel a cycle that leads to sterile inflammation and carcinogenesis, whereas in antigen-presenting cells, inflammasomes can stimulate anticancer immune responses. The inhibition of inflammasomes or neutralization of their products, mainly IL-1β, and IL-18, has profound effects on carcinogenesis and tumor progression [54].

Many other inflammasomes are able to cleave pro-IL-1β, including NALP3, AIM-2, NLRC4 (IPAF) and NLRP6, and for this it is not possible to discard the involvement of any of these other inflammasomes in the effects described here. Future studies to solve these questions are necessary.

In conclusion, although preliminary, our results show that stimulation with TNF-α is sufficient to trigger caspase-1 activation and IL-1β secretion, providing a mechanism for activation of the NALP1 inflammasome in SK-N-MC cells. Because both of them are main proinflammatory cytokine released in so many viral conditions, ischemia and stroke, it is reasonable to hypothesize that interfering with inflammasome activation may prove to be beneficial in delaying development of several neurodegenerative diseases in which the IL-1 inflammatory response plays a pathogenic role.

## Supporting Information

Figure S1 **SK-N-MC cells were stimulated 24 h with TNF-α (20 ng/ml).** Cell lysates were subjected to SDS-PAGE and immunoblotting with anti-NALP1 (top blot). As loading control, the blots were stripped and incubated with anti-α-tubulin (bottom blot). Extracts from K562 cells were used as positive control.
(TIF)

Figure S2 **Flow cytometric image of kinetic changes in [Ca^2+^]i after stimulation with TNF-α and A23187 (2 µM).** SK-N-MC alive cells were selected based on their light scatter properties and the arrow indicates the time point at which the stimuli were added. The fluorescence of the fluo4-Ca^2+^-complex was evaluated as the MFI per 30 s interval. The first interval prior to stimulation represents the baseline [Ca^2+^]i.
(TIF)
