# Supplementary figures and images for: TNF-Α May Mediate Inflammasome Activation in the Absence of Bacterial Infection in More than One Way
Source: PLoS One. 2013 Aug 7;8(8):e71477. doi: 10.1371/journal.pone.0071477 (PMC3737100; doi:10.1371/journal.pone.0071477)

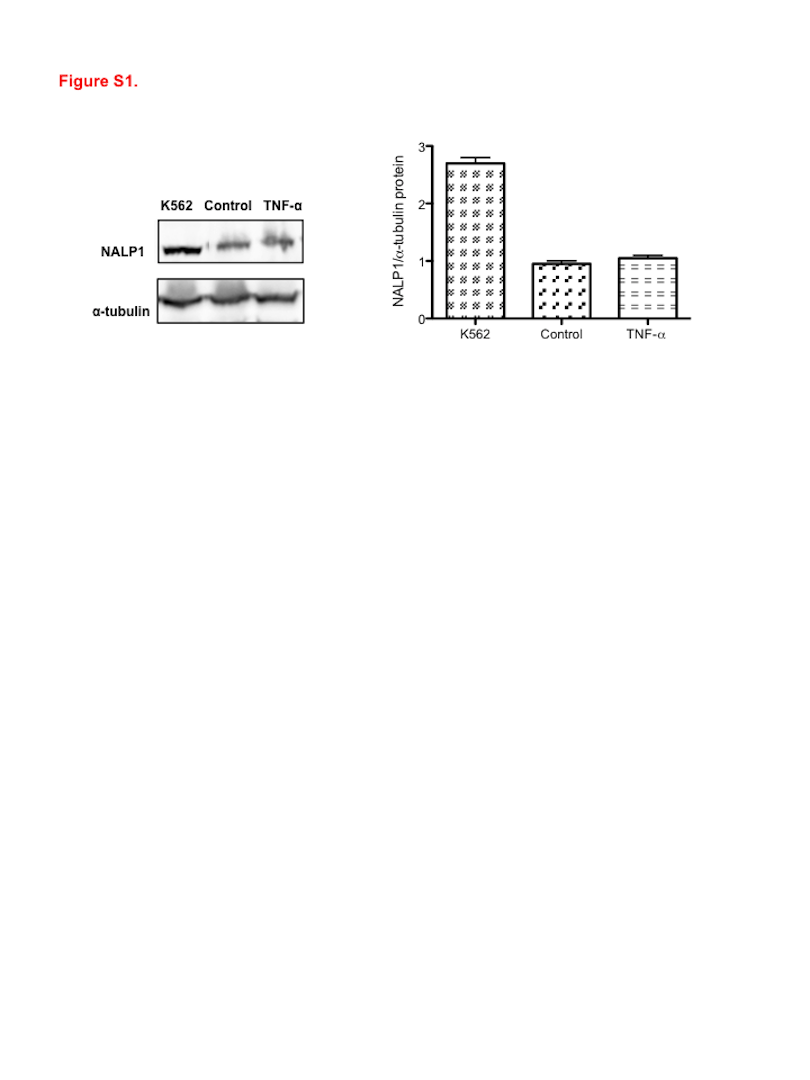

Supplement: Figure S1 — SK-N-MC cells were stimulated 24 h with TNF-α (20 ng/ml). Cell lysates were subjected to SDS-PAGE and immunoblotting with anti-NALP1 (top blot). As loading control, the blots were stripped and incubated with anti-α-tubulin (bottom blot). Extracts from K562 cells were used as positive control. (TIF) [file pone.0071477.s001.tif]

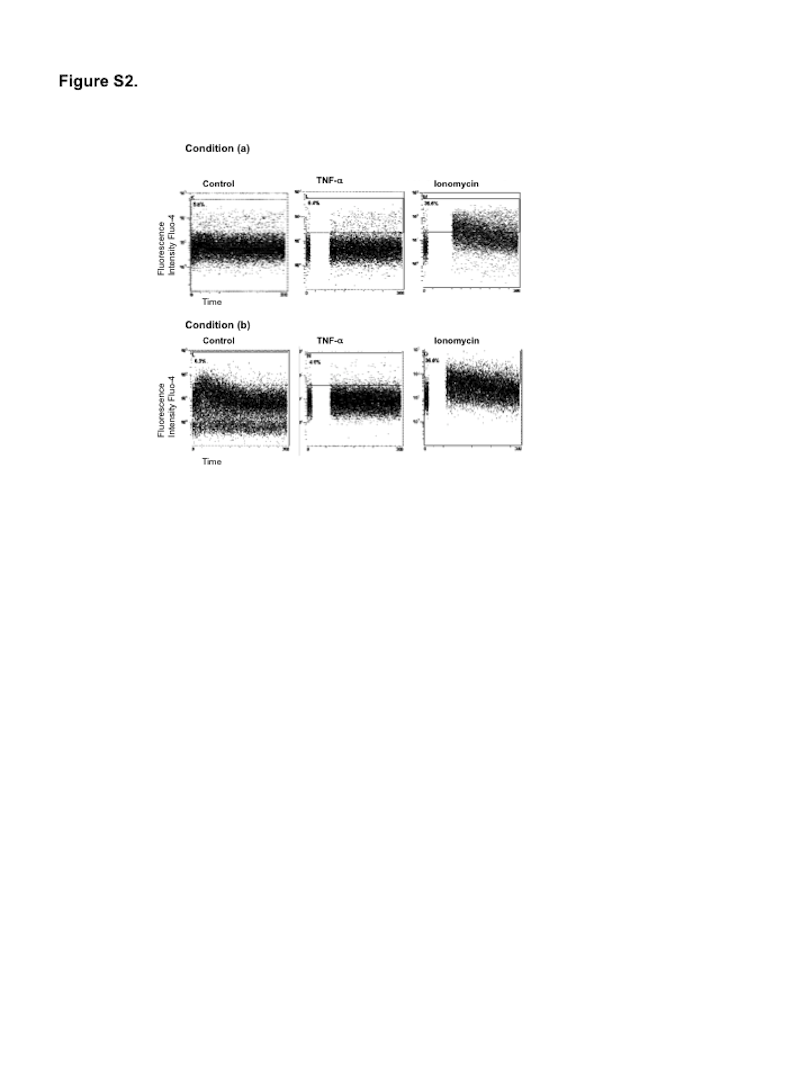

Supplement: Figure S2 — Flow cytometric image of kinetic changes in [Ca2+]i after stimulation with TNF-α and A23187 (2 µM). SK-N-MC alive cells were selected based on their light scatter properties and the arrow indicates the time point at which the stimuli were added. The fluorescence of the fluo4-Ca2+-complex was evaluated as the MFI per 30 s interval. The first interval prior to stimulation represents the baseline [Ca2+]i. (TIF) [file pone.0071477.s002.tif]
